# Structure of an essential bacterial protein YeaZ (TM0874) from *Thermotoga maritima* at 2.5 Å resolution

**DOI:** 10.1107/S1744309109022192

**Published:** 2009-10-27

**Authors:** Qingping Xu, Daniel McMullan, Lukasz Jaroszewski, S. Sri Krishna, Marc-André Elsliger, Andrew P. Yeh, Polat Abdubek, Tamara Astakhova, Herbert L. Axelrod, Dennis Carlton, Hsiu-Ju Chiu, Thomas Clayton, Lian Duan, Julie Feuerhelm, Joanna Grant, Gye Won Han, Kevin K. Jin, Heath E. Klock, Mark W. Knuth, Mitchell D. Miller, Andrew T. Morse, Edward Nigoghossian, Linda Okach, Silvya Oommachen, Jessica Paulsen, Ron Reyes, Christopher L. Rife, Henry van den Bedem, Keith O. Hodgson, John Wooley, Ashley M. Deacon, Adam Godzik, Scott A. Lesley, Ian A. Wilson

**Affiliations:** a Joint Center for Structural Genomics, http://www.jcsg.org, USA; b Stanford Synchrotron Radiation Lightsource, SLAC National Accelerator Laboratory, Menlo Park, CA, USA; c Protein Sciences Department, Genomics Institute of the Novartis Research Foundation, San Diego, CA, USA; d Center for Research in Biological Systems, University of California, San Diego, La Jolla, CA, USA; e Program on Bioinformatics and Systems Biology, Burnham Institute for Medical Research, La Jolla, CA, USA; f Department of Molecular Biology, The Scripps Research Institute, La Jolla, CA, USA; g Photon Science, SLAC National Accelerator Laboratory, Menlo Park, CA, USA

**Keywords:** YgjD, YeaZ, TM0874, essential genes, protein complexes

## Abstract

YeaZ is involved in a protein network that is essential for bacteria. The crystal structure of YeaZ from *Thermotoga maritima* was determined to 2.5 Å resolution. Although this protein belongs to a family of ancient actin-like ATPases, it appears that it has lost the ability to bind ATP since it lacks some key structural features that are important for interaction with ATP. A conserved surface was identified, supporting its role in the formation of protein complexes.

## Abstract 2

The crystal structure of an essential bacterial protein, YeaZ, from *T. maritima* identifies an interface that potentially mediates protein–protein interaction.

## 1. Introduction

*yeaZ* is an essential gene in many bacteria (Zhang & Lin, 2009 ▶), such as *Escherichia coli* (*yeaZ*), *Salmonella typhimurium* LT2 (*yeaZ*), *Bacillus subtilis* (*ydiC*), *Streptococcus pneumoniae* (*spr*0129), *Pseudo­monas aeruginosa* (PA14_16710) and *Francisella novicida* (FTN_1148). A genome-wide study of the *E. coli* interaction network revealed that YeaZ forms a complex with YgjD (Butland *et al.*, 2005 ▶). A recent study further demonstrated that *E. coli* YeaZ can interact with either YgjD or YjeE, with YgjD as the preferred partner, suggesting that YeaZ is part of a protein network that may be involved in DNA metabolism and cell division (Handford *et al.*, 2009 ▶). YgjD is homologous to Kae1 (kinase-associated endopeptidase 1), a com­ponent of the yeast KEOPS/EKC complex (kinase, endopeptidase and other proteins of small size/endopeptidase-like and kinase associated to transcribed chromatin) that is necessary for telomere maintenance and transcription of essential eukaryotic genes (Downey *et al.*, 2006 ▶; Kisseleva-Romanova *et al.*, 2006 ▶). Kae1 and its homologs belong to the ASKHA (acetate and sugar kinase/Hsp70/actin) superfamily (Mao *et al.*, 2008 ▶; Hecker *et al.*, 2007 ▶, 2008 ▶). Recent crystal structures of YeaZs from *E. coli* (EcYeaZ), *S. typhimurium* (StYeaZ; Jeudy *et al.*, 2005 ▶; Nichols *et al.*, 2006 ▶) and *Thermotoga maritima* (TmYeaZ; this study) indicate that YeaZ is structurally related to Kae1 and thus belongs to the same superfamily.

Here, we report the 2.5 Å resolution crystal structure of TmYeaZ from *T. maritima* (TM0874) in the light of current knowledge of the involvement of YeaZ in protein complexes, which was not available when the original StYeaZ structure was reported (Nichols *et al.*, 2006 ▶). The structure of TmYeaZ was determined using the high-throughput pipeline of the Joint Center for Structural Genomics (JCSG; Lesley *et al.*, 2002 ▶) as part of the National Institute of General Medical Sciences’ Protein Structure Initiative (PSI; http://www.nigms.nih.gov/Initiatives/PSI/). The *tm0874* gene of *T. maritima* encodes a protein with a molecular weight of 22 986 Da (residues 1–­206) and a calculated isoelectric point of 6.35.

## 2. Materials and methods

### 2.1. Protein production and crystallization

The gene encoding TmYeaZ (GenBank AAD35955.1; gi:4981408; Swiss-Prot Q9WZX7) was amplified by polymerase chain reaction (PCR) from *T. maritima* genomic DNA using *PfuTurbo* (Stratagene) and primers corresponding to the predicted 5′ and 3′ ends (forward primer, 5′-ATGAACGTTCTGGCACTCG-3′; reverse primer, 5′-CTCTTAATTAAGTCGCGTTAGCCCCTTTTCTTTTTTTCCCAG-3′). The PCR product was cloned into plasmid pMH4 (developed at the JCSG), which encodes an expression and purification tag (MGS­DKIHHHHHH) at the amino-terminus of the full-length protein. The cloning junctions were confirmed by DNA sequencing. Protein expression was performed in a selenomethionine-containing medium using *E. coli* strain GeneHogs (Invitrogen). Lysozyme was added to the culture at the end of fermentation to a final concentration of 250 µg ml^−1^ and the cells were harvested. After one freeze–thaw cycle, the cells were sonicated in lysis buffer [50 m*M* Tris–HCl pH 7.9, 50 m*M* NaCl, 10 m*M* imidazole, 1 m*M* tris(2-carboxyethyl)phos­phine hydrochloride (TCEP)] and the lysate was clarified by centrifugation at 32 500*g* for 30 min. The soluble fraction was applied to nickel-chelating resin (GE Healthcare) pre-equilibrated with lysis buffer, the resin was washed with wash buffer [50 m*M* Tris–HCl pH 7.9, 300 m*M* NaCl, 40 m*M* imidazole, 10%(*v*/*v*) glycerol, 1 m*M* TCEP] and the protein was eluted with elution buffer [20 m*M* Tris–HCl pH 7.9, 300 m*M* imidazole, 10%(*v*/*v*) glycerol, 1 m*M* TCEP]. The eluate was diluted tenfold to 45 ml with buffer *Q* [20 m*M* Tris–HCl pH 7.9, 5%(*v*/*v*) glycerol, 1 m*M* TCEP] containing 50 m*M* NaCl and loaded onto a 6 ml Resource Q column (GE Healthcare) pre-equilibrated with the same buffer. A linear gradient of 50–500 m*M* NaCl in buffer *Q* was used to elute the protein and the appropriate fractions were pooled. The protein was concentrated to 1 ml by centrifugal ultrafiltration (Millipore) and diluted to 15 ml with crystallization buffer (20 m*M* Tris–HCl pH 7.9, 150 m*M* NaCl, 1 m*M* TCEP). This process was repeated two more times, resulting in a 3375-fold buffer exchange. The protein was then concentrated to 14 mg ml^−1^ for crystallization, with its concentration being determined using Coomassie Plus Protein Assay Reagent (Pierce). To determine its oligomeric state, we analyzed TmYeaZ using a 1 × 30 cm Superdex 200 column (GE Healthcare) coupled with miniDAWN static light-scattering and Optilab differential refractive-index detectors (Wyatt Technology). The mobile phase consisted of 20 m*M* Tris–HCl pH 7.9, 150 m*M* NaCl and 0.02%(*w*/*v*) sodium azide. The molar mass was calculated using *ASTRA* 5.1.5 software (Wyatt Technology). The protein was crystallized using the nanodroplet vapor-diffusion method (Santarsiero *et al.*, 2002 ▶) with standard JCSG crystallization protocols (Lesley *et al.*, 2002 ▶). Sitting drops consisting of 200 nl protein solution and 200 nl crystallization reagent above a 50 µl reservoir were used. Initial screening for diffraction was carried out using the Stanford Automated Mounting system (SAM; Cohen *et al.*, 2002 ▶) at the Stanford Synchrotron Radiation Lightsource (SSRL; Menlo Park, California, USA). The crystal used for structure solution was obtained in 10%(*v*/*v*) 2-methyl-2,4-pentanediol (MPD) and 0.1 *M* citrate pH 4.0 at 277 K. A rectangular, plate-shaped crystal (∼50 × 30 × 15 µm) was harvested after 10 d. For cryoprotection, additional MPD was added to the crystal, bringing the final concentration to 25%(*v*/*v*). Diffraction images were indexed in space group *C*222.

### 2.2. Data collection, structure solution and refinement

Multi-wavelength anomalous diffraction (MAD) data were collected at SSRL on beamline 11-1 at wavelengths corresponding to the high-energy remote (λ_1_) and inflection (λ_2_) of a selenium MAD experiment. The data sets were collected at 100 K using an ADSC Q315 detector. The MAD data were integrated and reduced using *XDS* and then scaled using the program *XSCALE* (Kabsch, 1993 ▶). Selenium sites were located with *SHELXD* (Sheldrick, 2008 ▶) and refined using *autoSHARP* (Bricogne *et al.*, 2003 ▶). Phase refinement and automatic model building was performed with *RESOLVE* (Terwilliger, 2003 ▶). Model completion and refinement were per­formed with *Coot* (Emsley & Cowtan, 2004 ▶) and *REFMAC* (Winn *et al.*, 2003 ▶). Loose NCS restraints for both main chains and side chains (positional and thermal weights of 5.0 and 10.0, respectively) were applied between the two monomers. Each monomer was defined as a TLS group. Experimental MAD phases in the form of Hendrickson–Lattman coefficients were used as restraints during refinement. *CCP*4 programs were used for data conversion and other calculations (Collaborative Computational Project, Number 4, 1994 ▶). Data-processing and refinement statistics are summarized in Table 1 ▶.

### 2.3. Validation, deposition and figures

**Figure 1:**
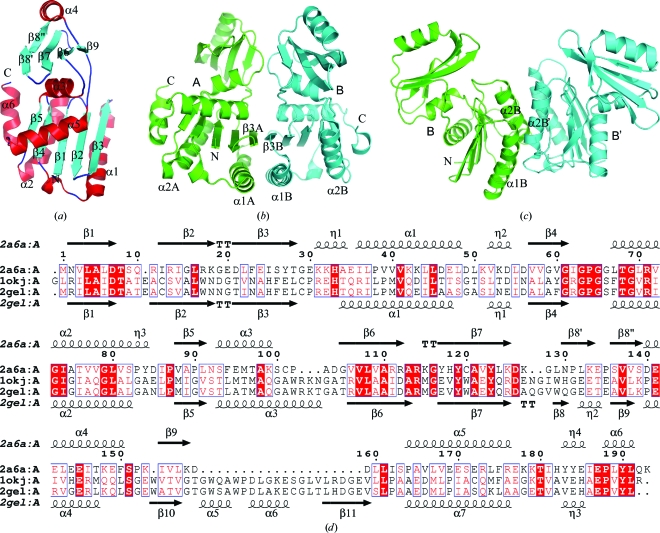
Crystal structure of TmYeaZ (TM0874) from *T. maritima*. (*a*) Ribbon diagram of TmYeaZ monomer. Helices α1–α6 and β-strands β1–β9 are labeled. (*b*) A dimer of TmYeaZ consisting of two protomers in the asymmetric unit. (*c*) Alternate dimer formed by the crystallographic twofold axis. (*d*) Sequence alignment between TmYeaZ (PDB code 2a6a), EcYeaZ (PDB code 1okj) and StYeaZ (PDB code 2gel). The secondary structure and sequence numbering of TmYeaZ are shown in the top row. The secondary structure of StYeaZ is shown in the bottom row.

The quality of the refined structure was analyzed using the *JCSG Quality Control* server, which verifies the stereochemical quality of the model using *AutoDepInputTool* (Yang *et al.*, 2004 ▶), *MolProbity* (Lovell *et al.*, 2003 ▶) and *WHATIF* 5.0 (Vriend, 1990 ▶), the agreement between the atomic model and the data using *SFCHECK* 4.0 (Vaguine *et al.*, 1999 ▶) and *RESOLVE* (Terwilliger, 2003 ▶), the protein sequence using *ClustalW* (Thompson *et al.*, 1994 ▶), the atomic occupancies using *MOLEMAN*2 (Kleywegt, 2000 ▶) and the consistency of NCS pairs. It also evaluates the difference in *R*_cryst_/*R*_free_, expected *R*_free_/*R*_cryst_ and maximum/minimum *B* values by parsing the refinement log file and PDB header. Analysis of the crystal packing was performed using the *PISA* server (Krissinel & Henrick, 2007 ▶). The sequences used for Fig. 3 are the top hits from a *BLAST* search (Altschul *et al.*, 1997 ▶) against the nonredundant protein-sequence database using TmYeaZ as a probe; only sequences with lengths between 180 and 250 residues were retained for the analysis (237 sequences). Multiple sequence alignments were performed using *MAFFT* (Katoh *et al.*, 2005 ▶). Mapping of sequence conservation onto the protein was performed by *CONSURF* (Landau *et al.*, 2005 ▶). Fig. 1(*d*) was generated using *ESPript* (Gouet *et al.*, 2003 ▶) with secondary structures assigned by *DSSP* (Kabsch & Sander, 1983 ▶). Fig. 3(*c*) was generated using *WEBLOGO* (Crooks *et al.*, 2004 ▶). All other figures were prepared with *PyMOL* (DeLano Scientific).

## 3. Results and discussion

The selenomethionine derivative of full-length TmYeaZ with an N-­terminal His tag was expressed in *E. coli* and purified by metal-affinity chromatography. The crystal structure of TmYeaZ was determined in space group *C*222 at 2.5 Å resolution using the MAD method. The final TmYeaZ model includes a dimer (residues 1–193 for chain *A* and 0–187 for chain *B* (residue 0 is the last residue of the His tag; the remainder of the His tag is disordered; Figs. 1 ▶ *a* and 1 ▶ *b*), two unknown ligands (UNL), which were modeled as discrete O atoms without geometry restraints, and 23 water molecules in the asymmetric unit. The two independent molecules in the asymmetric unit (*A*, *B*) are similar to each other, with a root-mean-square difference of 0.53 Å for 187 aligned C^α^ atoms. The C-termini (194–206 of chain *A* and 188–206 of chain *B*) were not modeled owing to a lack of interpretable electron density. The Matthews coefficient (*V*_M_; Matthews, 1968 ▶) for TmYeaZ is 3.25 Å^3^ Da^−1^ and the estimated solvent content is 61.9%. The Ramachandran plot produced by *MolProbity* shows that 95.5 and 99.5% of the residues are in favored and allowed regions, respectively. The two Ramachandran outliers (residues 115 and 151 of chain *A*) are located in regions of poor electron density. TmYeaZ is composed of nine β-strands (β1–­β9), six α-helices (α1–α6) and four 3_10_-helices (η1–η4). The total β-sheet, α-helical and 3_10_-helical content is 31.6, 32.6 and 6.2%, respectively.

The molecular weight of TmYeaZ in solution was determined to be 52 720 Da by analytical size-exclusion chromatography in combination with static light scattering. As a monomer of His-tagged SeMet-TmYeaZ would have a calculated molecular weight of 24 404 Da, it is likely that TmYeaZ exists as a dimer in solution. Analysis of the crystal packing suggests two possible modes of dimerization. In the first possibility (Fig. 1 ▶ *b*), the two independent monomers in the asymmetric unit would form a dimer (*AB* dimer) with a buried surface of 1240 Å^2^ per monomer (12.7% of the monomer surface area). The β3 strands from the two N-terminal domains pack together in an antiparallel manner to form an extended β-sheet. In the alternate mode, in which two dimers (*A*_2_ and *B*_2_) are formed by the crystallo­graphic twofold axis [Fig. 1 ▶ *c*, only the *B*_2_ (*B*–*B*′) dimer is shown], the α1 and α2 helices of their N-terminal domains are packed into a four-helix bundle, which generates a V-­shaped dimer. This dimer interface buries a surface area of 870 Å^2^ per monomer for the *B*_2_ dimer (9% of the *B* monomer surface area) and 565 Å^2^ per monomer for the *A*_2_ dimer (5.7% of the *A* monomer surface area). Although this second type of dimer interaction is weaker, it is interesting to note that a similar mode of dimerization is conserved in the crystal structures of both StYeaZ and EcYeaZ (Nichols *et al.*, 2006 ▶).

**Figure 2:**
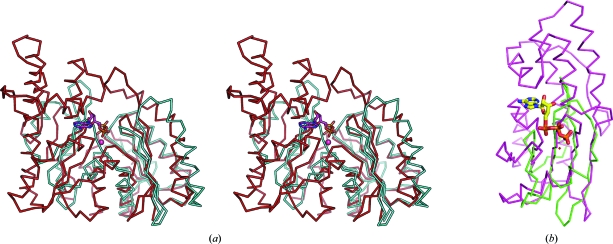
Structural comparisons of TmYeaZ and Kae1 (PDB code 2ivp). (*a*) Stereoview of the superposition of TmYeaZ (cyan) and Kae1 (red). (*b*) Superposition of the second domain of TmYeaZ (green) and the second domain of Kae1 (magenta). ATP bound to Kae1 is shown in stick representation.

TmYeaZ belongs to the actin-like ATPase superfamily, which typically contains a duplication of ribonuclease H-like domains (Andreeva *et al.*, 2004 ▶). In TmYeaZ, the first ribonuclease H-like domain is composed from both the N-terminus (residues 1–100) and the C-terminus (residues 160–193) of the protein. The second domain (residues 101–159) can be superimposed onto the N-terminal portion of the first domain with an r.m.s.d. of 3.18 Å for 47 aligned C^α^ atoms; however, it lacks the segment corresponding to the α1–β4 region of the first domain. The top hits from *DALI* (Holm & Sander, 1995 ▶) identified two bacterial YeaZ proteins: EcYeaZ (PDB code 1okj; *Z* = 21.0, r.m.s.d. = 2.1 Å for 188 aligned C^α^ atoms, 22% sequence identity; C. Abergel, S. Jeudy & J. M. Claverie, unpublished work) and StYeaZ (PDB code 2gel; *Z* = 20.6, r.m.s.d. = 2.3 Å for 187 aligned C^α^ atoms, 21% sequence identity; Nichols *et al.*, 2006 ▶). Since detailed structural comparisons of these YeaZ homologs and ASKHA proteins have been reported previously (Nichols *et al.*, 2006 ▶), we only briefly summarize the new results related to TmYeaZ. A structure-based sequence alignment of YeaZs is shown in Fig. 1 ▶(*d*). Compared with the other two YeaZs, TmYeaZ lacks the 20-residue αβ insertion between β9 and α5 in the second domain. Overall, the substantial structural similarities among these YeaZs suggest a common function. TmYeaZ also displays strong structural similarities to Kae1, especially in the first domain (PDB code 2ivp; r.m.s.d. = 2.1 Å for 110 aligned C^α^ atoms, 18% sequence identity; Hecker *et al.*, 2007 ▶). The second domain of Kae1 has an additional helical domain inserted between β8 and α4 of TmYeaZ, as well as an αβ insert between β9 and α5. Most strikingly, the orientation of the second domain of YeaZs with respect to the first domain differs significantly from that of Kae1 and other ASKHA proteins (Fig. 2 ▶ *a*; Hecker *et al.*, 2007 ▶; Nichols *et al.*, 2006 ▶). We were unable to identify a conserved ATP-binding site at the domain interface, although it has previously been proposed that YeaZ may still bind nucleotides through significant rearrangement of its two domains (Nichols *et al.*, 2006 ▶). YeaZs also lack the metal-binding motif of Kae1. In ASHKA proteins, the second domain plays an important role in stabilizing the adenosine base of the bound ATP. However, the substructure that interacts with the base in Kae1 is absent in TmYeaZ (Fig. 2 ▶ *b*). Thus, it is unclear how a suitable environment for nucleotide binding could be assembled within TmYeaZ.

**Figure 3:**
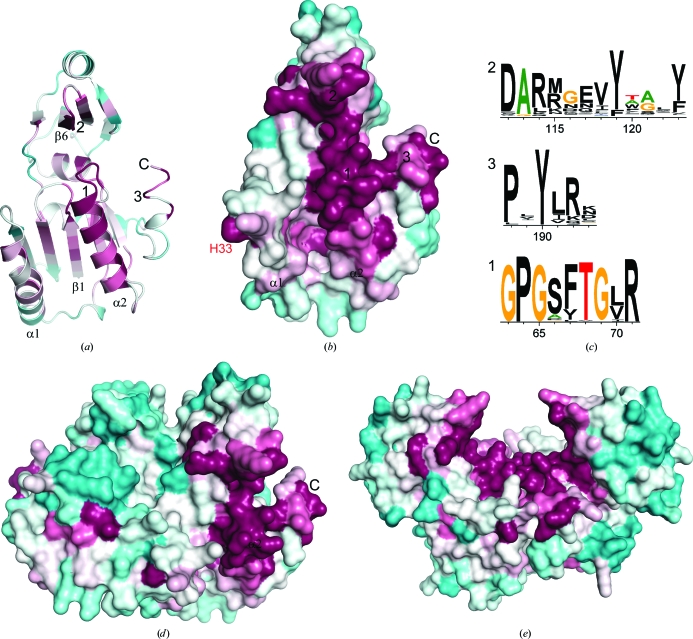
Mapping of conserved regions onto the TmYeaZ structure. (*a*) Ribbon representation of TmYeaZ colored by sequence conservation of 237 homologs of TmYeaZ. The most conserved residues are shown in magenta and the least conserved residues in cyan. The most conserved regions of the structure are marked 1, 2 and 3, respectively. (*b*) Molecular surface of TmYeaZ colored by sequence conservation. The orientation of the molecule is the same as in Fig. 2 ▶(*a*). (*c*) A sequence logo representation of the three most conserved regions in YeaZ. A logo consists of stacks of symbols, one stack for each position in the sequence. The overall height of the stack indicates the sequence conservation at that position, while the height of the symbols within the stack indicates the relative frequency of each amino or nucleic acid at that position. (*d*, *e*) Molecular surface of the *AB* (*d*) and *A*_2_ (or *B*_2_) (*e*) dimers colored by sequence conservation, as in Figs. 1(*b*) and 1(*c*).

EcYeaZ forms a stable complex with YgjD, but can also interact with the YjeE ATPase in a mutually exclusive manner (Handford *et al.*, 2009 ▶). In order to identify sites that are potentially important for TmYeaZ function, we studied the sequence-conservation pattern of the YeaZ and Kae1 families in the context of the YeaZ structures (Fig. 3 ▶). The most prominent common feature of these proteins is the prevalence of the sequence motif GPG*XX*TG*X*R located at the N-­terminus of a helix (α2 of TmYeaZ). This motif, which is reminiscent of a phosphate-binding motif, is close to the ATP-binding site in Kae1, but does not directly interact with ATP in Kae1. The arginine in this motif is exposed on the face of a helix in both Kae1 and YeaZs. Other conserved residues of the YeaZ homologs that are clustered around this motif include residues from the N-terminus of α1 (Lys32 and His33), the C-terminus of β1 (Asp7 and Thr8), the C-terminal portion of β6 (Arg112, Ala113 and Arg114), β7 (Tyr119) and the C-­terminus of α6 (Pro188, Tyr190 and Gln192). This cluster of conserved residues indicates that this region is likely to be directly involved in the function of YeaZ. In TmYeaZ, a prominent positively charged electrostatic surface overlaps with this conserved surface; however, this feature is not conserved in EcYeaZ or StYeaZ. We speculate that part of or the entire conserved surface is involved in mediating protein–protein interactions between YeaZ and YgjD (TM0145) or YjeE (TM1632) in *T. maritima*.

**Figure 4:**
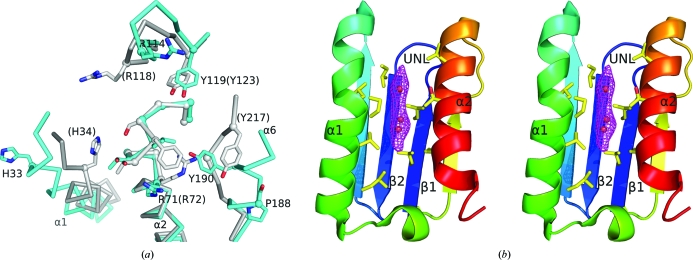
The conserved residues display large conformational flexibility. (*a*) Conserved residues on the potential binding surface of TmYeaZ (cyan) and StYeaZ (gray). The C^α^ positions of these residues are highlighted by spheres. The residues of StYeaZ are labeled in parentheses. (*b*) A stereoview of the unknown ligand (UNL; shown as red spheres) located between α1 and α2 of TmYeaZ (present in both monomers; the *B* monomer is shown here). The experimental density for the UNL (after solvent flattening and twofold averaging) is contoured at 1.5σ. Nearby protein residues are shown on stick representation.

It is worth noting that the conserved residues in α6 and α1 of TmYeaZ display large conformational differences compared with StYeaZ and EcYeaZ (Fig. 4 ▶ *a*). The residues corresponding to α6 of TmYeaZ are not in a helical conformation in StYeaZ and EcYeaZ despite being highly conserved in primary sequence. In the crystal structures of EcYeaZ and StYeaZ, α1 is packed more closely to α2 in an arrangement similar to that in Kae1. α1 and β2 are also stabilized by a hydrogen bond involving a conserved histidine (His34) from the N-­terminus of α1 and the β1–β2 loop and a potential disulfide bond between two cysteines (Cys13 and Cys30 in StYeaZ). A corresponding interaction was not observed in TmYeaZ, since α1 is more distant from α2 and, as a result, the conserved histidine (His33 in TmYeaZ) is exposed and further from the conserved cluster (Fig. 4 ▶ *a*). The wider gap between α1 and α2 in TmYeaZ partially exposes the hydrophobic interior of the β1 and β2 strands, which are buried in the other YeaZ structures. Interestingly, a section of unaccounted-for electron density was observed in the resulting exposed groove of TmYeaZ and was modeled as an unidentified ligand (UNL; Fig. 4 ▶ *b*). This UNL could be a lipid or MPD, but it was not possible to unambiguously identify its nature. As the UNL is close to the conserved surface identified above, it may have functional implications; an alternative explanation that cannot be ruled out at present is that it is a crystallization artifact. Overall, the large conformational differences in these conserved residues may indicate that they are flexible in solution and may adopt a more rigid conformation when TmYeaZ is bound in the complex.

One of the dimers observed in the crystal lattice is likely to have physiological significance. EcYeaZ also dimerizes (Handford *et al.*, 2009 ▶). However, it is not clear from the crystal structure which one of the two possible dimers represents the physiologically relevant form. The mode of dimerization affects the placement and exposure of the conserved surface identified above. The conserved surfaces of each monomer are fully exposed at either end in the *AB* dimer, while they are clustered together in the *A*_2_ (or *B*_2_) dimer (Figs. 3 ▶ *d* and 3 ▶ *e*). The *A*_2_ dimer imposes very strict steric restrictions on the size of and the mode of interaction with its partners. Based on the size of interface, the *AB* dimer may be more stable. Given that the first domains of YgjD and YeaZ are structurally similar, the observed crystal-packing inter­action seen in the *A*_2_ dimer may mimic the complex between YeaZ and YgjD.

The specific function of YeaZ is still unknown. In Gram-positive organisms such as *B. subtilis*, YdiB (the YjeE homolog), YdiC (the YeaZ homolog), YdiD and YdiE (the Kae1 or Gcp homolog) are located within the same operon. The four corresponding proteins in *T. maritima* [TM1632 (TmYjeE), TM0874 (TmYeaZ), TM0577 and TM0145 (TmYgjD)] are dispersed across the genome. It has been suggested that YeaZ mediates the proteolysis of YgjD (Handford *et al.*, 2009 ▶). However, no active site mimicking those of known peptidases can be identified based on the crystal structure. Thus, its mechanism remains unclear. YeaZ may fulfill its function by contributing one or more critical functional groups to a bipartite active site in a heterodimeric complex with YgjD or YjeE. Since YgjD and YjeE are both ATPases, it is possible that YeaZ functions as an ATPase inhibitor or activator. Further experiments are needed to elucidate the function of YeaZ and its possible partners.

## 4. Conclusions

The crystal structure of TmYeaZ (TM0874), a YeaZ homolog that is an essential protein in bacteria, has been elucidated. Based on its structure, TmYeaZ by itself is not likely to be an ATPase owing to the absence of a clearly defined ATP-binding site. A potential interface for mediating protein–protein interaction was identified. It remains possible that TmYeaZ may play a role in nucleotide binding or hydrolysis in a complex involving YgjD.

Essential gene products are excellent targets for antibacterial drugs. Unlike Kae1, YeaZ could be a potential drug target since it only seems to be present in bacteria. The crystal structure and the information presented here should be valuable for further biochemical characterization of this important bacterial protein. Additional information about TmYeaZ is available from TOPSAN (Krishna *et al.*, 2010 ▶)  http://www.topsan.org/explore?PDBid=2a6a.

## Supplementary Material

PDB reference: TM0874 from *T. maritima*, 2a6a, r2a6asf

## Figures and Tables

**Table 1:** Summary of crystal parameters, data collection and refinement statistics for TmYeaZ (PDB code 2a6a) Values in parentheses are for the highest resolution shell.

	λ_1_ MADSe	λ_2_ MADSe
Space group	*C*222	
Unit-cell parameters (Å)	*a* = 93.27, *b* = 217.11, *c* = 51.95	
Data collection		
Wavelength (Å)	0.9184	0.9794
Resolution range (Å)	29.2–2.50 (2.64–2.50)	29.2–2.58 (2.72–2.58)
No. of observations	66857	60100
No. of reflections	18685	16886
Completeness (%)	99.5 (99.9)	98.7 (93.0)
Mean *I*/σ(*I*)	12.3 (2.3)	12.7 (2.5)
*R*_merge_ on *I*†	0.06 (0.55)	0.06 (0.48)
Model and refinement statistics		
Resolution range (Å)	29.2–2.5	
No. of reflections (total)	18685	
No. of reflections (test)	955	
Completeness (%)	99.5	
Data set used in refinement	λ_1_ MADSe	
Cutoff criterion	|*F*| > 0	
*R*_cryst_‡	0.191	
*R*_free_§	0.235	
Stereochemical parameters		
Restraints (r.m.s. observed)		
Bond lengths (Å)	0.018	
Bond angles (°)	1.69	
Average isotropic *B* value (Å^2^)	62.7¶	
ESU†† based on *R*_free_ value (Å)	0.24	
Protein residues/atoms	381/2889	
Water molecules/ligands	23/2	

† *R*_merge_ = 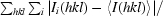 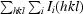.

‡ *R*_cryst_ = 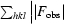 − 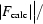 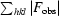, where *F*_calc_ and *F*_obs_ are the calculated and observed structure-factor amplitudes, respectively.

§ *R*_free_ is the same as *R*_cryst_ but for 5.0% of the total reflections chosen at random and omitted from refinement.

¶ This value represents the total *B* that includes TLS and residual *B* components.

†† Estimated overall coordinate error (Cruickshank, 1999 ▶).
